# Validation and reliability testing of the Breast-Q latissimus dorsi questionnaire: cross-cultural adaptation and psychometric properties in a Swedish population

**DOI:** 10.1186/s12955-021-01812-x

**Published:** 2021-07-03

**Authors:** Lynne Kamya, Emma Hansson, Linn Weick, Emma Hansson

**Affiliations:** Department of Plastic Surgery, University of Gothenburg, The Sahlgrenska Academy, Institute of Clinical Sciences, Sahlgrenska University Hospital, Gröna Stråket 8, 413 45 Gothenburg, Sweden

**Keywords:** Breast reconstruction, Latissimus dorsi flap, Patient-reported outcomes measures, Psychometrics, Validity, Reliability, Health-related quality of life

## Abstract

**Background:**

The main aim of post-mastectomy breast reconstruction is to improve the patient’s quality of life, which makes high-quality and validated patient-reported outcome measurements essential. None of the established instruments include evaluation of donor-site morbidity, such as impact on upper extremity and back function, when a latissimus dorsi (LD) muscle is used; and BREAST-Q LD questionnaire was therefore recently developed for this purpose. The aim of this study was to translate into Swedish and culturally adapt the BREAST-Q LD questionnaire’s two subscales, appearance and function, and perform a psychometric evaluation of the subscales in a Swedish population of patients.

**Methods:**

This was a cross-sectional study. The questionnaire was translated according to established guidelines. The questionnaires were sent to all patients operated using an LD flap between 2007 and 2017. Internal consistency was assessed using Cronbach’s α. Inter-item correlations and corrected item-total correlations were calculated using the Pearson’s correlation coefficient. Convergent validity was evaluated by comparing the BREAST-Q LD questionnaire to the Western Ontario Osteoarthritis of the Shoulder Index, using the Spearman correlation coefficient. Test–retest reliability was tested with intraclass correlation coefficients (ICCs), and the coefficient of variation and Bland–Altman plots were drawn. Floor and ceiling effects were calculated. Known-group validation was tested by comparing scores from the patients and from normal controls using the Mann–Whitney *U*-test and by calculating eta squared effect size.

**Results:**

The questionnaires were sent to 176 eligible patients and 125 responded (71%). The patients had been operated a mean of 6.6 years ago, and most (92%) had previous radiation. Internal consistency was satisfactory for both subscales. The correlation coefficients between questions were *r* > 0.30 for all items of both scales. The corrected item-total correlation coefficient ranged from 0.62 to 0.90. As hypothesised, the function scale was correlated with the WOOS “Physical symptoms” subscale. Reliability was adequate according to the ICCs. The ceiling effect threshold for the appearance scale was reached and that for the back scale was almost reached. There were significant differences between patients and controls, in the hypothesised direction.

**Conclusions:**

The results of this study support a good internal consistency, convergent validity, test–retest reliability and known-group validation for the Swedish BREAST-Q LD questionnaire. However, it may be difficult to discriminate between patients with very mild and those with no symptoms using the appearance scale.

*Trial registration*: ClinicalTrials.Gov identifier NCT04526561.

## Background

The main aim of post-mastectomy breast reconstruction is to increase the patient’s health-related quality of life (HRQoL) and restore her body image [1], which makes high-quality and validated patient-reported outcome measurements (PROMs) essential to allow for comparison between methods [2]. In recent years, a number of validated tools have been developed for this purpose [3], of which one of the most frequently used is the BREAST-Q [4, 5]. The original BREAST-Q reconstruction questionnaire contains three satisfaction domains: Satisfaction with breast, Satisfaction with overall outcome, and Satisfaction with process of care, and three well-being domains: Psychological well-being, Physical well-being, and Sexual well-being [4]. The instrument includes questions on complications and consequences of implants and of donor-site morbidity of abdominally based flaps [4]. However, as donor-site morbidity of other types of reconstructions are lacking, the BREAST-Q latissimus dorsi (LD) questionnaire was recently developed [6] as a complement to the general BREAST-Q reconstruction module.

Breast reconstruction using a LD musculocutaneous flap was first described in the beginning of the twentieth century, [7] and is still a commonly used method globally [8], as it is considered a safe option with a reliable and good result and low donor-site morbidity [8]. Nonetheless, harvesting the LD muscle might have an impact on upper extremity and back function; [9–13] the assessment of donor-site morbidity is therefore fundamental in PROMs evaluating the results of breast reconstructing with a pedicled LD flap. Indeed, there are very few long-term studies evaluating the donor-site effects after breast reconstruction with an LD flap [9, 12].

The aim of the present study was to translate into Swedish and culturally adapt two BREAST-Q LD questionnaire subscales: the Satisfaction with back appearance scale and the Satisfaction with back and shoulder function scale, and perform a psychometric evaluation of the questionnaire in a Swedish population of patients reconstructed with an LD flap. Psychometric properties were assessed on the basis of reliability and validity.

## Methods

### Study design and protocol

This was a cross-sectional study to validate a PROM questionnaire for breast reconstruction using LD in a Swedish population. It is one of the studies described in the Reconstruction with back donor-site flaps study protocol (ClinicalTrials.Gov identifier NCT04526561).

### Ethics

Permission to translate and validate the LD modules of the BREAST-Q questionnaire was granted by the Mapi Research Trust (Lyon, France). Use of the BREAST-Q, authored by Drs. Klassen, Pusic, and Cano, was made under license from Memorial Sloan Kettering Cancer Center (New York, USA).  The Regional Ethical Committee of Gothenburg (Gothenburg, Sweden) reviewed and approved the study (254-18). Procedures followed were in accordance with the Helsinki Declaration. All participants provided written informed consent to participate in the study and to publication.

### Setting

The study was performed in the Department for Plastic and Reconstructive Surgery, at Sahlgrenska University Hospital in Gothenburg, one of seven university hospitals in Sweden. Around 350–400 breast reconstructions, of which about 50 are autologous, are performed every year at the department.

### Questionnaires

The BREAST-Q LD questionnaire includes two scales: Satisfaction with back appearance, with 8 questions (items) and Satisfaction with back and shoulder function, with 11 questions, asking patients to rate how often they have been bothered by problems during the last 2 weeks on a five-point scale ranging from ‘none of the time’ to ‘all of the time’ [6]. The items of the scales were developed using a qualitative methodology in the United States [6], and was subsequently validated in a British population [6], resulting in an 8-item Satisfaction with back appearance scale and an 11-item Satisfaction with back and shoulder function scale.

The scales were validated [6] using the Rasch measurement model, generating a conversion table in which sum scores of the scales (8–40 [14] and 11–55 [15], respectively) were converted to equivalent Rasch transformed scores (0–100). A higher score represents a better outcome [6]. Both scales had good internal consistency (Cronbach’s α = 0.95 and 0.94, respectively) and high corrected item-total correlations (range 0.75–0.86 and 0.61–0.83, respectively). The Person Separation Indices were acceptable (0.80 and 0.86, respectively). The authors calculated distribution-based minimally important differences of 11 and 9.15 points, respectively [6]. Some aspects of the BREAST-Q LD questionnaire, such as floor/ceiling effects and test–retest reliability, have never been investigated.

The scales were recorded according to the BREAST-Q users’ manual [14, 15]; that is, “None of the time” = 5, “A little of the time” = 4, “Some of the time” = 3, “Most of the time” = 2, “All of the time” = 1. The mean of the completed questions was inserted if missing data were less than 50% of the questions of the scale. In the control group of healthy women, ‘1’ was inserted in cases where the participants had not answered the questions on back scar appearance. The original conversion tables were utilised to convert the raw scale summed score into an equivalent Rasch transformed score [14, 15].

The Western Ontario Osteoarthritis of the Shoulder Index (WOOS) is a PROM which measures HRQoL in people with osteoarthritis of the shoulder [16]. The WOOS has four subscales: Physical symptoms; Sport, recreation, and work; Lifestyle; and Emotions. The items are composed of visual analogue scales of 100 mm, where zero equals no symptoms. The scores are added to give a total score of a maximum of 1900. Zero equals no symptoms. Responses are sometimes given as percentages. WOOS scores correlated to other scales measuring similar constructs, such as the University of California at Los Angeles (UCLA) shoulder rating scale (*r* = 0.63) [16] and the Shoulder Rating Questionnaire (*r* = 0.83) [17]. A good reliability has been demonstrated for the total score (ICC 0.96) and for the subscales (ICC 0.87–0.95) and the instrument has a good responsiveness (standardised response mean 1.9 for the English version [16] and 1.02 for the Swedish version [17]). The instrument has been validated for Sweden [17].

### Translation process

**Fig. 1:**
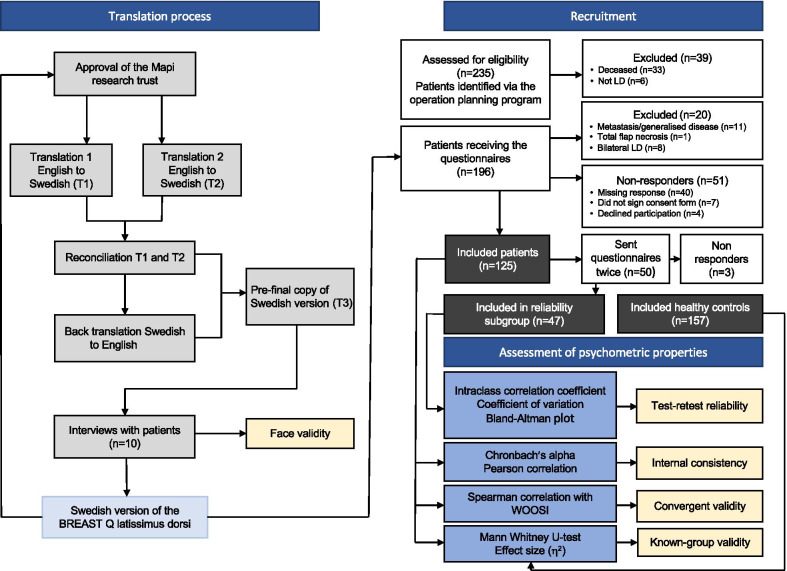
The course of the study. The figure was inspired by figure 1 in Zmnako and Chalabi. Cross-cultural adaptation, reliability and validity of the Vertigo Symptom Scale—Short Form in the central Kurdish dialect. Health and Quality of Life Outcomes (2019) 17:125. Figure created by Åsa Bell, medical photographer, Department of Plastic and Reconstructive surgery, Sahlgrenska University Hospital, Gothenburg, Sweden

The questionnaire was translated according to established guidelines [18, 19]. Two independent translations from the English original of the BREAST-Q LD questionnaire into Swedish were performed by professional Swedish mother tongue translators, specialised in medicine. The researchers in the Department of Plastic and Reconstructive Surgery then created a single Swedish version. Discrepancies were discussed until consensus was reached. A back-translation from Swedish to English was performed by a professional English mother tongue translator, specialised in medicine. There are no item definitions to guide the translation of the BREAST-Q questionnaire. The authors of the original BREAST-Q LD questionnaire reviewed the back-translated version to ensure that the meaning of the items was equivalent to that of the original. A pilot test of the translated version was performed in five women waiting for a breast reconstruction with an LD flap (ages 43, 47, 56, 62, and 53 years) and five previously reconstructed women (ages 42, 47, 48, 65, and 59 years). All of the women were native speakers of Swedish. They were interviewed by a specially trained research nurse, who has worked with breast reconstruction patients for more than 30 years. A semi-structured interview guide on how the participants understood the questionnaires and interpreted the items was used (face validity), and if they found the items acceptable. A report was sent to the Mapi Research Trust who approved it. The process is summarised in Fig. 1.

### Participants, sample size, and data collection

Patients were identified through an operation planning programme*.* The questionnaire was sent to women who had had a breast reconstruction coded as an LD flap in the 2007–2017 operation planning programme. The sample size was based on the number of patients operated during this pre-specified time period; hence, a convenience sampling technique was used. A 10-year time period was chosen to allow for a sample meeting the minimum recommendations for validations studies, usually ranging from 50 to 200 [20].

The patients were sent an envelope including information about the study, a consent form, and the questionnaires to be answered. A stamped reply envelope was attached. Two remainders were sent after 2 and 4 weeks, in case the participant had not returned the questionnaire. The first fifty patients who answered the questionnaires, and fulfilled the inclusion criteria, were sent the questionnaires again 2 weeks after the first questionnaire so that a test–retest reliability analysis could be performed.

When a patient had consented to participation in the study, clinical background data were collected from the patients’ charts and eligibility for inclusion checked, after which the patient was included. Inclusion criteria were women > 18 years of age who had had a unilateral breast reconstruction with an LD flap. Exclusion criteria were relapse or metastatic disease, inability to give informed consent, insufficient Swedish language skills, total flap loss, and bilateral LD flaps. Women who had had bilateral LD flaps were excluded, as some of the questions in the BREAST-Q LD questionnaires are appropriate only for those who had undergone breast reconstruction only on one side; for example, ‘How often have you experienced weakness in your arm?’.

To obtain scores from healthy women, the questionnaire was sent to a thousand randomly selected women aged 18–80 in the Västra Götaland Region. The individuals’ addresses were obtained from the *Statens personadressregister, SPAR*, which includes all residents in Sweden.

### Psychometric evaluation: statistical analyses and hypotheses

Continuous variables were described by mean (standard deviation) and median (minimum and maximum). All tests were two-tailed and a *p* value of 0.05 was considered to indicate a statistically significant result. Statistical tests were performed using SAS software, version 9.4 (SAS Institute Inc, Cary, NC, USA) and SPSS, version 27 for Mac (IBM Corp, Armonk, NY, USA). The analyses are summarised in Fig. 1.

The Rasch analyses were not repeated, as that could have resulted in conversion tables that differ from the original conversion tables [14, 15], complicating comparisons of surgical outcomes between different countries.

Internal consistency measures indicate how the different questions (items) are correlated, that is, if these measure the same concept (construct) and if combining scores into a single score is therefore justified [21]. Internal consistency was assessed using Cronbach’s α [22] for the two scales. Alpha values ranging from 0.70 to 0.95 are often considered acceptable [23]. A low Cronbach’s α means that there is a lack of correlation between the questions of the scale, and that it is therefore unjustified to combine these into a total score. A very high Cronbach’s α (≥ 0.95) could indicate that there is a redundancy of questions in the scale [21]. Inter-item correlations and corrected item-total correlations were calculated using Pearson’s correlation coefficient (*r*). The inter-item correlation indicates the extent to which the questions of the scales were related within the two scales, and a *r* value of between 0.2 and 0.8 is considered to indicate a good consistency. Higher correlations could indicate that some items are too similar, and therefore redundant. Corrected item-total correlations are correlations between the scores from that item with the average scores of the other items. The corrected item-total correlations should be *r* ≥ 0.3 [24].

Convergent validity measures indicate how two tools, such as two questionnaires, that are theoretically related are actually related [21]. The BREAST-Q LD scale Satisfaction with back and shoulder function score was correlated with the WOOS Physical symptoms subscale. The Spearman correlation coefficient (ρ) was calculated. Correlation between the Satisfaction with back and shoulder function scale and the WOOS Physical symptoms subscale should be strong (ρ > 0.70), as these measure similar constructs with similar approaches.

In the original validation [6], the author defined a distribution-based minimally important difference as 0.5 of a standard deviation (SD). In this study, we used the same definition for the minimally detectable change (MDC) [25], that is, the smallest detectable changes that are not caused by measurement errors or random errors.

Test–retest reliability indicates the degree to which repeated measurements in stable patients produce similar scores [21]. Sometimes called longitudinal reproducibility [26], test–retest reliability was investigated by inviting a subgroup of fifty participants answer the questionnaire on two separate occasions, with an interval of 2 weeks between the measurements. Intraclass correlation coefficients (ICCs) were calculated [27] to assess agreement between the two measurements. ICCs can range from 0 to 1, where 1 corresponds to complete agreement; that is, there is no measurement error. An ICC of < 0.5 was assumed to indicate poor reliability, ≥ 0.5 to ≤ 0.75 moderate, > 0.75 to ≤ 0.9 good, and > 0.9 excellent, as suggested by Koo and Li [28]. The coefficient of variation was calculated as (intra-individual SD/mean) × 100. Bland–Altman plots [29] of the individuals’ two separate scores were drawn. The direction of the mean difference should be close to zero, and the limits of agreements should ideally be less than the MDC.

Floor and ceiling effects were calculated as the percentage of participants who obtained the minimum and the maximum scores, that is 100 and 0 points. The threshold was considered met if more than 15% of the patients achieved the minimum or maximum scores [30].

Known-group validation was tested by comparing patient scores and scores from normal controls using the Mann–Whitney *U*-test and by calculating the eta squared effect size (η^2^ = Z^2^/n − 1). Effect sizes of 0.01 should be interpreted as small, 0.06 as moderate, and 0.14 as large, according to Cohen [31]. We hypothesised that normal controls would score significantly higher than patients, and that the effect size was large.

## Results

### Translation and pilot testing

Two main issues were examined in order to reconcile the two Swedish translations. The first was the translation of the word *satisfaction* in scale titles. One of the translators suggested *belåtenhet* and the other *nöjdhet*. *Nöjdhet* was chosen as this is the most common expression in modern, spoken Swedish and as *patient-reported satisfaction* is translated as *patientrapporterad nöjdhet* in Swedish. The other issue was the expression of the genitive in Swedish. One of the translators kept the translations close to the English version; for example, *the length of the scar* was translated as *längden på ärret*. It was decided that the other suggestion, *ärrets längd*, was more idiomatic in Swedish.

None of the interviewed women who had filled out the questionnaire had any difficulty in understanding the questions and interpreting them correctly, and none of them suggested any alternative solutions. The women found all of the items acceptable. Therefore, the face validity was considered adequate. Pilot testing did not lead to any linguistic changes in the instrument.

### Response rates and participant characteristics

The questionnaires were sent to 196 patients, of which 20 did not fulfil the inclusion criteria and were excluded, leaving 176 eligible patients (Fig. 1). The response rate was 71% (125/176). None of the patients operated in 2007–2009 answered the questionnaire. In addition, the BREAST-Q LD was sent to 1000 healthy women, of which 157 responded (16%). Demographics are given in Table 1. An analysis of possible differences between respondents and non-respondents could not be performed, as the non-responders did not consent to chart review.

**Table 1:** Demographics

	Patients	Controls
	Mean (SD) Median (range) or N (%)	Mean (SD) Median (range) or N (%)
Age at time of surgery, years	53 (9.5)	
	53 (32–75)	
Age at time of questionnaire, years	60 (9.9)	52 (17)
	60 (38–81)	52 (18–74)
Time since LD, years	6.6 (2.2)	
	7 (3–10 years)	
Indication for LD		
Previous radiation	115 (92%)	
Salvage procedure	10 (8%)	
Denervation		
Missing data	88 (70%)	
Yes	24 (19%)	
No	13 (10%)	
Satisfaction with back appearance	75.5 (24)	93 (13)
	74 (0–100)	100 (47–100)
Minimally detectable change (MDC)	12	6.5
Satisfaction with back and shoulder function	61.6 (21)	76 (19)
	57 (0–100)	75 (33–100)
Minimally detectable change (MDC)	10.5	9.5

### Data completeness

For the Satisfaction with appearance scale, the answers to seven questions were complete. One answer was missing for one question for both measurements 1 and 2. Answers to seven questions on the Satisfaction with back function, were complete. One answer was missing for four questions for measurement 1 and no answers were missing for measurement 2. In the control group, none of the women had answered the four questions on the appearance of the back scar in the Satisfaction with appearance scale, and as the participants did not undergo surgery ‘1’ was inserted. There were no missing data for the Satisfaction with back function in the control group.

### Internal consistency

Internal consistency of both subscales was satisfactory. Cronbach’s α was 0.96 for Satisfaction with back appearance, and 0.95 for Satisfaction with back and shoulder function. The α values were not affected by the removal of any items.

The inter-item correlation for all items of both scales was *r* > 0.30 (Tables 2, 3). For the appearance scale, the correlation coefficient (*r*) was > 0.80 between question 2 (‘How often have you been bothered by the length of your scar?’) and two other questions―question 1 (‘How often have you been bothered by the location of your back scar?’) and question 7 (‘How often have you been bothered by how your scar looks?’); between question 6 (‘How often have you been bothered by the shape (contour) of your back?’) and two other questions―question 4 (‘How often have you been bothered by the sides of your back not matching?’) and question 5 (‘How often have you been bothered by how your back looks); and between question 7 (‘How often have you been bothered by how your back scar looks?’) and question 8 (‘How often have you been bothered by having to wear certain clothes in order to hide your back scar?’). For the function scale, the correlation coefficient (*r*) was > 0.80 for question 1 (‘How often have you experienced shoulder stiffness?’) and question 2 (‘How often have you experienced shoulder pain?’); and question 4 (‘How often have you experienced difficulty doing activities with your arms above your head?’) and 5 (‘How often have you experienced difficulty doing activities with your arms outstretched?’).

**Table 2:** The inter-item correlations (*r*) of the Satisfaction with back appearance scale

	Q^a^	Q^b^	Q^c^	Q^d^	Q^e^	Q^f^	Q^g^	Q^h^
Q^a^	1.00							
Q^b^	0.82*	1.00						
Q^c^	0.65*	0.74*	1.00					
Q^d^	0.71*	0.69*	0.71*	1.00				
Q^e^	0.74*	0.71*	0.74*	0.90*	1.00			
Q^f^	0.70*	0.64*	0.66*	0.87*	0.91*	1.00		
Q^g^	0.75*	0.83*	0.77*	0.75*	0.78*	0.70*	1.00	
Q^h^	0.71*	0.80*	0.67*	0.71*	0.73*	0.64*	0.86*	1.00

*Q* question

*p < 0.0001

^a^Location of back scar

^b^Length of back scar

^c^Noticeability of back scar to others

^d^Match of the sides of the back

^e^Look of  back

^f^The shape (contour) of  back

^g^Look of  back scar

^h^Wearing certain clothes in order to hide back scar

**Table 3:** The inter-item correlations (*r*) of the Satisfaction with back and shoulder function scale

	Q^a^	Q^b^	Q^c^	Q^d^	Q^e^	Q^f^	Q^g^	Q^h^	Q^i^	Q^j^	Q^k^
Q^a^	1.0										
Q^b^	0.84*	1.0									
Q^c^	0.60*	0.62*	1.0								
Q^d^	0.52*	0.40*	0.55*	1.0							
Q^e^	0.50*	0.42*	0.60*	0.85*	1.0						
Q^f^	0.50*	0.49*	0.56*	0.69*	0.74*	1.0					
Q^g^	0.61*	0.53*	0.72*	0.75*	0.79*	0.79*	1.0				
Q^h^	0.58*	0.46*	0.58*	0.59*	0.67*	0.63*	0.67*	1.0			
Q^i^	0.54*	0.48*	0.65*	0.56*	0.64*	0.58*	0.68*	0.80*	1.0		
Q^j^	0.48*	0.43*	0.43*	0.73*	0.75*	0.70*	0.74*	0.70*	0.73*	1.0	
Q^k^	0.48*	0.50*	0.50*	0.67*	0.72*	0.80*	0.76*	0.57*	0.60*	0.73*	1.0

*Q* question

*p < 0.0001

^a^Shoulder stiffness

^b^Shoulder pain

^c^Back pain

^d^Difficulty doing activities, arms above head

^e^Difficulty doing activities, arms outstretched

^f^Weakness in arm

^g^Difficulty, repeat use of shoulder/back muscles

^h^Tightness when you stretch your arm

^i^A pulling feeling in back

^j^Difficulty reaching for objects

^k^Difficulty carrying heavy objects

The corrected item-total correlation coefficient (*r*) ranged from 0.79 to 0.90 for the appearance scale and from 0.62 to 0.88 for the function scale (Table 4); the corrected item-total correlations were therefore considered acceptable.

**Table 4:** Corrected-item total correlation (*r*)

	Q^a^	Q^b^	Q^c^	Q^d^	Q^e^	Q^f^	Q^g^	Q^h^	Q^i^	Q^j^	Q^k^
Appearance scale	0.82	0.84	0.79	0.86	0.90	0.83	0.88	0.83			
Function scale	0.69	0.63	0.74	0.78	0.82	0.80	0.88	0.76	0.77	0.81	0.80

*Q* question

### Convergent validity

The Satisfaction with shoulder and back function scale was correlated with the WOOS Physical symptoms subscale (**ρ** = 0.69, *p* < 0.001). However, the correlation coefficient was 0.01 lower than the a priori hypothesis of 0.70.

### Test–retest reliability

**Fig. 2:**
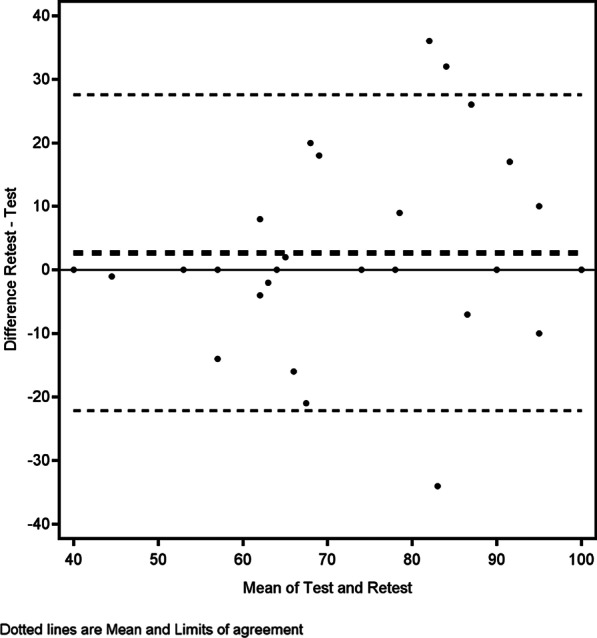
Bland–Altman plot for the satisfaction with back appearance scale

**Fig. 3:**
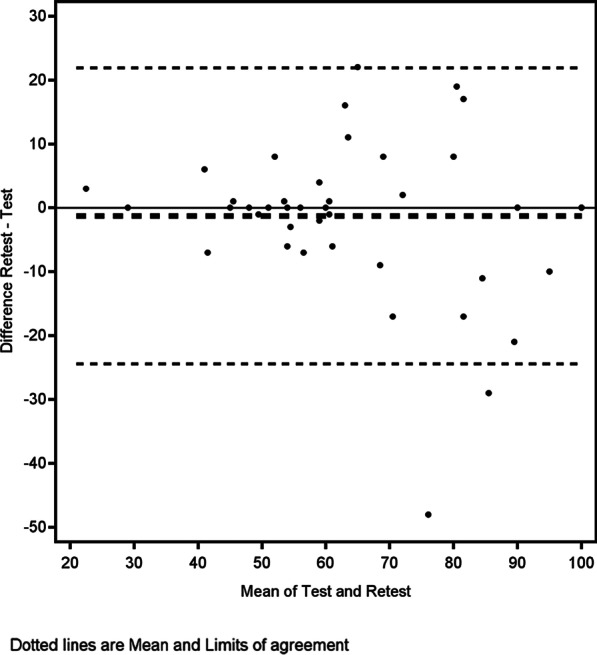
Bland–Altman plot for the satisfaction with shoulder and back function scale

None of the patients had any surgery between measurement 1 and measurement 2. The mean difference between score 1 and score 2 was 2.7 (SD 13, *p* = 0.15) for the Satisfaction with back appearance scale and – 1.28 (SD 12) for the Satisfaction with back and shoulder function. The ICC of the patients’ two scores were 0.77 for the appearance scale and 0.84 for the function scale. Hence, reliability, according to the ICC, was good for the appearance scale and excellent for the function scale. The coefficient of variation was 11% for the appearance scale and 12% for the function scale. According to the Bland–Altman plots (Figs. 2, 3), the overall assessment of the comparisons of score 1 and score 2 shows that the direction of the mean difference is close to zero, and the limits of agreements are greater than the MDCs for both scales.

### Floor and ceiling effects

On the Satisfaction with back appearance scale, 46 patients (37%) obtained the maximum score and one patient (0.8%) the minimum score. On the Satisfaction with back and shoulder function scale, 18 patients (14%) obtained the maximum score and one patient (0.8%) the minimum score. Hence, the ceiling effect threshold for the appearance scale was reached, while that for the back scale was and almost reached.

For the functional scale, 39% of the controls hit the ceiling and 0 hit the floor; for the function scale, 30% of the participants hit the ceiling and 0 hit the floor. Hence, the ceiling effect threshold for both scales was researched.

### Known-group validation

There were significant differences between patients and controls in the hypothesised direction for both the Satisfaction with back appearance scale (*p* < 0.001) and for the Satisfaction with back and shoulder function scale (*p* < 0.001). The mean total scores on the appearance scale in controls were 88.8 (SD 19.3) and 76.4 (SD 19.3) for the function scale. Hence, the differences between patients and controls (respectively 13.3 and 14.8) were greater than the predefined MDCs. The effect size (η^2^) was 0.17 for the appearance scale and 0.13 for the function scale.

## Discussion

This is a linguistical and psychometric validation study of the the Satisfaction with back appearance scale and the Satisfaction with back and shoulder function scale of the BREAST-Q LD questionnaire for Sweden.

The Cronbach’s α values of the Swedish scales were similar to the values found in the original British validation [6] (respectively 0.96 vs. 0.95) [6] for Satisfaction with back appearance and [6] for Satisfaction with back and shoulder function (respectively 0.95 vs. 0.94). In the present study, there was a correlation coefficient of > 80 between some questions in each scale (inter-item correlation), which could indicate a redundancy of questions. However, a certain redundancy is preferable over a further reduction of items that would complicate comparisons between outcomes from different countries. In the original validation [6], the corrected item-total correlation range was 0.75–0.86 for the appearance scale and 0.61–0.83 for the function scale, which is similar to ranges found for the Swedish scales (0.79–0.90 and 0.62–0.88, respectively). The internal consistency of the Swedish BREAST-Q LD questionnaire may therefore be considered good and on par with the original English version.

There is no golden standard for measuring patient-reported back and shoulder function. In the present study, a validated scale measuring function in people with osteoarthritis of the shoulder [17] was chosen. Given that the scale is constructed for people with osteoarthritis of the shoulder and the lack of a gold standard, a ρ value of 0.69 may be considered acceptable, although it did not reach the hypothesised ρ value of > 0.70. The lack of convergent validation of the appearance scale is a limitation of the present study. To our knowledge, there is no other validated PROMs measuring back appearance and scarring. Nonetheless, another comparator could have been employed, such as an in-house constructed visual analogue scale. The convergent validity of the Swedish function scale is good, but further studies are needed to examine the convergent validity of the Swedish appearance scale. The convergent validity of the original English scale has not been published [6].

According to the ICC, the test–retest reliabilities of the scales are good and excellent, respectively. Nonetheless, it is noteworthy that the Bland–Altman plots demonstrated that the difference between the first and second measurements sometimes exceeded that of the predefined MDCs for the scales. The implied MDCs of the British and the Swedish validations are similar, 11 [6] versus 12 for the appearance scale and 9.2 [6] versus 10.5 for the function scale; these can therefore be considered fairly accurate. Nonetheless, the minimally detectable change is based on the statistical characteristics of the sample and should not be confounded with the minimally important difference (MID), that is, change in score that constitutes a clinically meaningful effect that can be used, for example, to balance benefits and harms and cost-effectiveness of a certain treatment. MIDs are better estimated with anchor-based methods that examine the relation between PROM scores and other measures that are interpretable and relevant to the patient [25]. Moreover, the changes between score 1 and score 2 could represent both a true clinical change between the two measurements and a measurement error. Theoretically, the patients’ satisfaction with their back appearance should not change very much over a period of 2 weeks in patients who were operated several years ago. Even so, satisfaction with appearance is a very subjective measurement that might fluctuate [32], which could explain the difference. The back and shoulder function is affected by aspects other than their operation, leading to a change in score. Moreover, the negative phrasing of the questions in the instrument could encourage the patient to focus on certain aspects of the condition and not on others [33, 34]. The effect of this when PROMs are used to measure satisfaction with breast reconstruction has never been studied. The test–retest reliability of the original English scale was not tested in the previous validation study [6]. In summary, the test–retest reliability of the Swedish BREAST-Q LD questionnaire is adequate according to established criteria [21], and on par with other BREAST-Q modules [4]. Nonetheless, more data on the stability of measurements performed with the instrument are required, especially in relation to the responsiveness to true change [35].

The ceiling effect of the appearance scale (37%) was reached and that of the function scale (14%) was almost reached, which makes it likely that some items could be missing from the lower end of the scales, indicating limited content validity [21]. The ceiling effect suggests that patients with a few symptoms cannot be distinguished from patients with no symptoms. Similarly, the Person Separation Index test that was performed during the original scale development [6] suggested that it could be difficult to discriminate between patients with very mild and those with no symptoms using the appearance scale. This is further strengthened by the fact that the ceiling effects of the appearance scale were similar in patients and controls in this study (37% vs. 30%). However, the clinical importance of being able to discriminate between patients with very mild symptoms and no symptoms is unclear. The floor and ceiling effects of the original English scales have never been published [6]. Further studies are needed to analyse if this reduced sensitivity has any practical implications. To date, no pre- and post-operative studies in the same cohort have been published.

Despite the fact that the scales cannot be used to differentiate between back and shoulder problems caused by the reconstruction and problems of other aetiologies and that the scale might not be able to discriminate between mild and no symptoms, the BREAST-Q LD questionnaire seems to be able to distinguish operated patients from controls. In the original validation of the English scales [6], it was hypothesised that patients who were operated with a completely autologous LD flap would have more functional problems than women who were operated with an LD flap in combination with an implant, and that that women who had had a perioperative complication at the donor site would have a lower score than women who had not. Differences could be seen in both groups in the hypothesised direction, but these differences were less than the predefined MDC. Hence, the known-group validation of the English scale is somewhat unclear [6], which is common when a priori groups are used [36]. The original scale has never been tested in healthy controls. The known-group validity of the Swedish BREAST-Q LD questionnaire seems to be adequate.

A prerequisite for using PROMs to evaluate the effect of treatment is that the instrument is responsive, that is, it can detect clinically relevant changes over time [21]. The responsiveness could not be tested with the present study design. It has not been tested in the previous validation study either [6]. The same scales are supposed to be used both pre-operatively and post-operatively [14, 15] to evaluate the effect of surgery, but to our knowledge, no study giving pre-operative values of the questionnaire has yet been published. We detected a number of weaknesses of using the questionnaire in non-operated patients when the questionnaire was used in the control group. For example, many of the controls had not answered the questions on their back scar, as they did not have one. This is a potential problem if the scale is going to be used pre-operatively. We suggest that in cases when the questionnaire is used pre-operatively the answer “None of the time” is used as default response for questions a–c, e and g–h in the appearance scale; or that only the function scale is used pre-operatively. Further responsiveness testing of the BREAST-Q LD is needed.

The present study has a few limitations, including that the sample size was limited by patient availability and convenience sampling. The ‘rule of thumb’ for sample size in validation studies is to ensure a certain ratio of the number of participants to number of items (usually 3–10) and minimum recommendations, often ranging from 50 to 200 [20]. This would imply that our sample should have included between 57 and 190 patients; a sample size of 125 participants thus seems adequate, and this is one of the largest cohorts of LD flap reconstructions published in a Scandinavian setting. Moreover, sociodemographic factors were similar to those of previous studies on LD flap reconstruction [9–13] and the response rate was relatively high, indicating that the sample could be representative of the target population. In addition, the data completeness was comprehensive, further strengthening the validity of the results.

## Conclusions

The results of this study support a good internal consistency, convergent validity, test–retest reliability and known-group validation of the satisfaction with appearance scale and the satisfaction with back and shoulder function scale of the Swedish BREAST-Q LD questionnaire. However, it might be difficult to discriminate between patients with very mild and those with no symptoms using the appearance scale. Further responsiveness testing is needed for the BREAST-Q LD questionnaire. Additionally, anchor-based minimally important differences need to be established.

## Data Availability

The datasets generated and analysed during the current study are not publicly available due to patient confidentiality, but are available from the corresponding author on reasonable request and permissions.

## Abbreviations

η^2^: Eta squared
HRQoL: Health-related quality of life
ICC: Intraclass correlation coefficient
LD: Latissimus dorsi
MDC: Minimally detectable change
PROM: Patient-reported outcome measurement
*r*: Pearson’s correlation coefficient
ρ: Spearman correlation coefficient
SD: Standard deviation
WOOS: Western Ontario Osteoarthritis of the Shoulder Index
